# Cross-cultural adaptation and validation of the lebanese arabic version of the BACS scale (the brief assessment of cognition in schizophrenia) among stable schizophrenic inpatients

**DOI:** 10.1192/j.eurpsy.2021.1405

**Published:** 2021-08-13

**Authors:** C. El Haddad, P. Salameh, S. Hallit, S. Obeid, G. Haddad, J.-P. Clément, B. Calvet

**Affiliations:** 1 Research, Psychiatric Hospital of the Cross, Beirut, Lebanon; 2 Faculty Of Pharmacy / Medicine, Lebanese University, Beirut, Lebanon; 3 Faculty Of Medicine And Medical Sciences, Holy Spirit University of Kaslik (USEK), Beirut, Lebanon; 4 Faculty Of Arts And Sciences, Holy Spirit University of Kaslik (USEK), Beirut, Lebanon; 5 Pôle Universitaire De Psychiatrie De L’adulte Et De La Personne âgée, centre hospitalier Esquirol, Limoges, France; 6 Old Age Psychiatry, CHU de Limoges, Limoges, France

**Keywords:** schizophrénia, BACS, Arabic, cognition

## Abstract

**Introduction:**

The assessment of cognitive disorders in schizophrenia is becoming a part of clinical and research practice by using batteries that differ widely in their content. The Brief Assessment of Cognition in Schizophrenia (BACS) was developed to cover the main cognitive deficits of schizophrenia.

**Objectives:**

The objective of this study was to assess concurrent validity of the Arabic version of the BACS with a standard neurocognitive battery of tests in Lebanese patients with schizophrenia and healthy controls.

**Methods:**

A sample of 120 stable inpatients diagnosed with schizophrenia and 60 healthy controls received the Arabic version of the BACS in a first session, and a standard battery in a second session.

**Results:**

The mean duration of completion for the BACS was 31.2 ± 5.4 min in patients with schizophrenia. All tests demonstrated significant differences between controls and patients (p<0.01). A principal components analysis demonstrated that a one-factor solution best fits our dataset (64.8% of the variance). A high Cronbach alpha was found (0.85). The BACS composite scores were significantly correlated with the standard battery composite scores in patients (r=0.78, p < 0.001) and healthy controls (r=0.77, p < 0.001). Also, the correlation analysis between the BACS sub-scores and the standard battery sub-scores showed significant results (p < 0.05). The Arabic-BACS demonstrated high ability to discriminate patients with schizophrenia from healthy controls.

**Conclusions:**

The results showed that the Arabic version of the BACS is a useful tool for assessing cognition in patients with schizophrenia and could be used in clinical practice in Lebanon.

